# Potential treatment of COVID-19 by inhibitors of human dihydroorotate dehydrogenase

**DOI:** 10.1007/s13238-020-00769-9

**Published:** 2020-08-06

**Authors:** Yechun Xu, Hualiang Jiang

**Affiliations:** 1 CAS Key Laboratory of Receptor Research, Shanghai Institute of Materia Medica, Chinese Academy of Sciences, Shanghai, 201203 China; 2 University of Chinese Academy of Sciences, Beijing, 100049 China; 3 Shanghai Institute for Advanced Immunochemical Studies and School of Life Science and Technology, ShanghaiTech University, Shanghai, 201210 China

The ongoing pandemic of severe acute respiratory syndrome coronavirus 2 (SARS-CoV-2), referred to as coronavirus disease 2019 (COVID-19), has caused over 13 million infections and over 560,000 deaths worldwide (https://www.who.int/emergencies/diseases/novel-coronavirus-2019/situation-reports), posing a significant threat to globally public health and economics. At present, no efficacious antiviral drugs and vaccines have been approved for the prophylaxis or treatment of COVID-19. Tremendous efforts have been made to develop drug and vaccine against SARS-CoV-2. The main protease (Mpro, also called 3CLpro) is an attractive drug target among coronaviruses, and several potent inhibitors of the SARS-CoV-2 3CLpro together with their crystal structures in complex with the protease have been reported (Dai et al., 2020; Jin et al., 2020; Zhang et al., 2020). While the viral RNA-dependent RNA polymerases (RdRp) are well-known broad-spectrum antiviral drug targets, the cryo-electron microscopy structures of the SARS-CoV-2 RdRp and its complex with remdesivir, a promising antiviral candidate developed by Gilead Sciences, validated the efficient inhibition of the viral RNA replication by remdesivir and provided a rational template for drug design to combat SARS-CoV-2 infections (Gao et al., 2020; Wang et al., 2020; Yin et al., 2020). In addition, the trimeric spike protein on the surface of SARS-CoV-2 plays a pivotal role during the viral entry by binding to the peptidase domain of angiotensin-converting enzyme 2 (ACE2), a host cell receptor (Yan et al., 2020). It has been revealed that not only the receptor binding domain which is recognized by ACE2 but also the N-terminal domain of the SARS-CoV-2 spike protein is targeting sites for therapeutic monoclonal antibodies (Chi et al., 2020). Accordingly, both the inhibitors of 3CLpro or RdRp and the antibodies targeting the spike protein provide potential candidates for development of the direct-acting antiviral (DAA) drugs for the treatment of COVID-19.

In addition to DAA drugs, host-targeting antiviral (HTA) agents, targeting host proteins required for the viral infection and replication, have advantages in overcoming drug resistance and combating a broad spectrum of viruses including the newly emerging virus (Ji and Li, 2020). Maraviroc, an antagonist of chemokine receptor type 5 for HIV treatment, presents a typical HTA drug. In a remarkable study published in this journal, Xiong et al. reported novel and potent inhibitors of human dihydroorotate dehydrogenase (DHODH) as broad-spectrum antiviral agents against RNA viruses including SARS-CoV-2 (Xiong et al., 2020).

**Figure 1:**
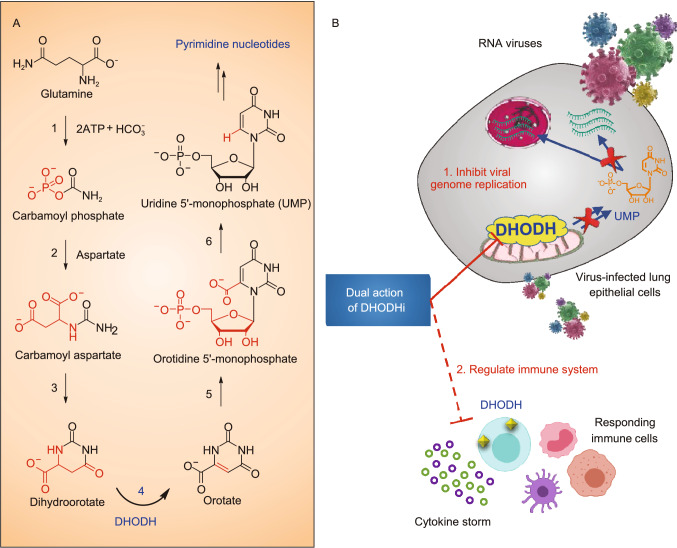
**DHODH in the** ***de novo*** **pyrimidine biosynthesis pathway and dual action of DHODHi**. (A) DHODH catalyzes the fourth step in the *de novo* pyrimidine biosynthesis pathway. (B) DHODH inhibitors (DHODHi) are broad-spectrum antivirals against RNA viruses with the dual action of inhibiting viral genome replication and regulating the immune system

Pyrimidines serve as crucial building blocks for the biosynthesis of DNA, RNA, phospholipids, and glycoproteins, which is essential for the cell survival as well as proliferation (Loffler et al., 2005). Human DHODH belongs to the class 2 DHODH family and is a flavin-dependent mitochondrial enzyme catalyzing the oxidation of dihydroorotate to orotate, the fourth step also a rate limiting step in the *de novo* biosynthesis of pyrimidine-based nucleotides (Reis et al., 2017) (Fig. 1A). By consequence, DHODH is an attractive therapeutic target for multiple diseases including cancer and autoimmune diseases (Lolli et al., 2018; Boschi et al., 2019; Madak et al., 2019). Leflunomide and its metabolite teriflunomide, and brequinar are well-known DHODH inhibitors and were evaluated in clinical trials (Lolli et al., 2018). Leflunomide was approved for the therapy of rheumatoid arthritis many years ago (Herrmann et al., 2000).

With a computer-aided hit discovery and optimization strategy, Xiong et al. identified two novel and potent inhibitors of DHODH with a thiazole scaffold, S312 and S416 (Diao et al., 2012; Li et al., 2015; Zhu et al., 2015). The IC_50_s of these two compounds against human DHODH were 29.2 and 7.5 nmol/L, respectively, a >10-fold increase in activity relative to the FDA-approved teriflunomide (IC_50_ = 307.1 nmol/L). The X-ray crystal structure of DHODH in complex with S416 also revealed the binding mode of two inhibitors at the ubiquinone-binding site of the enzyme. Moreover, two inhibitors exhibited significant antiviral activities against influenza A (H1N1, H3N2 and H9N2), Zika, Ebola, and SARS-CoV-2 in cells infected with various tested viruses, demonstrating that DHODH inhibitors possess broad-spectrum antiviral activity by interfering the *de novo* pyrimidine synthesis pathway. Low toxicities of the inhibitors suggest that the reduced production of pyrimidine restricts virus replication but not cell growth. Most notably, the EC_50_ of S416 against the viral replication in the cells infected with SARS-CoV-2 at MOI of 0.05 is 17 nmol/L, and the resulting selectivity index (SI = CC_50_/EC_50_) reaches 10 505.88. It is much more potent than that of teriflunomide or brequinar and is also by far the most effective inhibitor against SARS-CoV-2 in cells.

Another striking feature of this work is that S312 exhibited *in vivo* anti-influenza efficacy equivalent to that of oseltamivir, a marketed drug for the treatment of influenza. S312 at a dose of 5 mg/kg was also able to rescue all the influenza-infected mice from body weight loss and death. By contrast, previous studies often showed that inhibitors of either DHODH or the *de novo* pyrimidine biosynthesis pathway were ineffective against infection in animal models. In addition, the combination administration of S312 and oseltamivir resulted in 100% protection of the infected mice, superior to the single use of S312 or oseltamivir. S312 was also effective in the mice infected with an oseltamivir-resistant virus and had a remarkable advantage over oseltamivir to treat the late phase of the infectious disease. These results together demonstrated the feasibility of DHODH inhibitors used as efficacious antivirals as well as the combination of the DHODH inhibitor with DAA to overcome drug resistance.

As leflunomide and teriflunomide are used to treat autoimmune diseases such as rheumatoid arthritis and multiple sclerosis by regulating lymphocytes and the release of cytokines and chemokines, it is reasonable to conjecture that S312 and S416 would have the similar efficacy too. As anticipated, the combination use of S312 and oseltamivir significantly reduced the levels of IL-6, MCP-1, IL-5, KC/GRO (CXCL1), IL-2, IFN-γ, IP-10, IL-9, TNF-α, GM-CSF, EPO, IL-12p70, MIP-3α, and IL-17A/F in the animal model. Therefore, the DHODH inhibitors not only inhibit the viral replication but also have regulatory roles in cytokine/chemokine production. Cytokine storm frequently occurred with patients suffered from virus infections such as SARS-CoV and SARS-CoV-2, antiviral treatment alone is thereby not enough and should be combined with appropriate anti-inflammatory treatment. The DHODH inhibitors provide the ideal candidate to take both into consideration.

Taken together, this elegant work uncovers that DHODH is an attractive host target for developing broad-spectrum antivirals which achieve the efficacy through dual mechanism of action of antiviral and immuno-regulation (Fig. 1B), providing more therapeutic options in response to COVID-19 as well as other emergent RNA virus infections. In the present situation, S312 and S416, two potent inhibitors of DHODH with favorable drug-likeness and pharmacokinetic profiles, serve as right HTAs for further evaluation of therapeutic potential in COVID-19 treatment. Meanwhile, as a new concept for the treatment of COVID-19, the clinical trial of leflunomide has been initiated in England and founded by LifeArc (DEFEAT-COVID study) (https://www.lifearc.org/funding/covid-19-funding/).
